# HBV Reactivation in Patients Treated with Antitumor Necrosis Factor-Alpha (TNF-*α*) Agents for Rheumatic and Dermatologic Conditions: A Systematic Review and Meta-Analysis

**DOI:** 10.1155/2014/926836

**Published:** 2014-07-07

**Authors:** Fabrizio Cantini, Stefania Boccia, Delia Goletti, Florenzo Iannone, Emanuele Leoncini, Nikola Panic, Francesca Prignano, Giovanni Battista Gaeta

**Affiliations:** ^1^Division of Rheumatology, Misericordia e Dolce Hospital, Via Cavour 87/89, 59100 Prato, Italy; ^2^Section of Hygiene, Institute of Public Health, Department of Public Health, Università Cattolica del Sacro Cuore, L.go F.Vito 1, 00168 Rome, Italy; ^3^Translational Research Unit, Department of Epidemiology and Preclinical Research, INMI, Via Portuense 292, 00149 Rome, Italy; ^4^Rheumatology Unit, Interdisciplinary Department of Medicine, University of Bari, Piazza Giulio Cesare 1, 70124 Bari, Italy; ^5^Dermatology Clinic/ASF Department of Surgery and Translational Medicine, University of Florence, Via Lorenzo Il Magnifico 104, 50129 Florence, Italy; ^6^Section of Infectious Diseases, Department of Internal Medicine, Second University of Naples, Via S. Pansini 5, 80131 Naples, Italy

## Abstract

*Introduction.* Antitumor necrosis factor-alpha (TNF-*α*) agents are widely used for treatment of rheumatic and dermatological diseases. We conducted the systematic review and meta-analysis to assess the prevalence of HBV reactivation among patients treated with anti-TNF-*α*. *Methods and Findings.* A comprehensive literature search of MEDLINE, Scopus, and ISI Web of Knowledge databases was conducted. From 21 studies included in the systematic review, 9 included patients with occult chronic HBV infection and 6 included patients with overt infection while 6 addressed both groups. Based on 10 studies eligible for meta-analysis we report pooled estimate of HBV reactivation of 4.2% (95% CI: 1.4–8.2%, *I* ^2^: 74.7%). The pooled prevalence of reactivation was 3.0% (95% CI: 0.6–7.2, *I* ^2^: 77.1%) for patients with occult infection, and 15.4% (95% CI: 1.2–41.2%, *I* ^2^: 79.9%) for overt infection. The prevalence of reactivation was 3.9% (95% CI: 1.1–8.4%, *I* ^2^: 51.1%) for treatment with etanercept and 4.6% (95% CI: 0.5–12.5%, *I* ^2^: 28.7%) for adalimumab. For subgroup of patients without any antiviral prophylaxis the pooled reactivation was 4.0% (95% CI: 1.2–8.3%, *I* ^2^: 75.6%). *Conclusion.* Although HBV reactivation rate is relatively low in patients treated with anti-TNF-*α* for rheumatic and dermatological conditions, the antiviral prophylaxis would be recommended in patients with overt chronic HBV infection.

## 1. Introduction

Antitumor necrosis factor-alpha (TNF-*α*) agents are widely used for effective treatment of autoimmune rheumatic and dermatological diseases such as rheumatoid arthritis (RA), ankylosing spondylitis (SA), psoriasis (Ps), or psoriatic arthritis (PsA). Nevertheless, anti-TNF-*α* agents have been associated with growing number of adverse events, particularly infections [1, 2] of which some can be life threatening.

TNF-*α* is an important proinflammatory cytokine in the host defense mechanism against many intracellular pathogens. It suppresses hepatitis B virus (HBV) replication and promotes HBV eradication by stimulating HBV-specific cytotoxic T-cell response [3–5]. It has been reported that reactivation of HBV infection may occur directly due to lack of TNF-*α* or indirectly via diminishing T-cell activation [6, 7]. TNF-*α* inhibitors are therefore likely to induce HBV replication and reactivation in cases when chronic infection is present.

HBV is regarded as a leading cause of acute hepatitis, cirrhosis, and hepatocellular carcinoma [8], being responsible for about 600000 deaths every year [8]. Chronic HBV infection is defined as an overt when hepatitis B surface antigen (HBsAg) is detectable in the serum. Patients who present antibodies to hepatitis B core antigen (anti-HBc) with concurrent HBsAg negativity do not have chronic hepatitis but only experienced HBV infection and were able to clear it. Nevertheless, some of these patients may be occult carriers, harboring intrahepatic HBV replication [9], and therefore can be susceptible to HBV reactivation. Hepatitis B virus (HBV) reactivation in patients treated with anti-TNF-*α* agents has been frequently reported in the last decade, with inconsistent results [10]. Considering the high socioeconomic burden of HBV infection related conditions, as well as increasing role of anti-TNF-*α* agents in treatment of autoimmune rheumatic and dermatological diseases, it is highly important to estimate the impact of anti-TNF-*α* agents to HBV reactivation in these patients.

We have conducted a systematic review and meta-analysis in order to assess the prevalence of HBV reactivation among patients treated with anti-TNF-*α* agents because of RA, SA, Ps, and PsA.

## 2. Methods

This systematic review and meta-analysis have been reported following the PRISMA statement [21].

### 2.1. Search Strategy

We conducted comprehensive literature search of MEDLINE, Scopus, and ISI Web of Knowledge databases using the following search: ([infliximab] OR [rituximab] OR [etanercept] OR [adalimumab] OR [abatacept] OR [“anti-TNF”]) AND [“HBV reactivation”]. The search was limited to human subjects with language restriction to English studies until 1st September 2013. The snowball strategy, including manual search of the references listed by studies retrieved from the online databases and from previously published systematic reviews, was also performed to identify potential additional studies. Abstracts, systematic reviews, editorials, and case reports were not included.

### 2.2. Inclusion and Exclusion Criteria

The eligibility criteria for inclusion in the review implied that (i) patients should be affected by at least one of the following diseases: rheumatoid arthritis (RA), ankylosing spondylitis (SA), psoriasis (Ps), or psoriatic arthritis (PsA); (ii) study must refer to treatment with one or more of the following biologic agents: infliximab, rituximab, etanercept, adalimumab, and abatacept; (iii) the HBV serological status of patients prior to the pharmacological treatment and the prevalence of HBV reactivation after the treatment should be reported. Studies were excluded if it included only hepatitis C virus (HCV) infected patients (other than those also coinfected with HBV).

### 2.3. Data Extraction and Outcome Definition

Data from the included studies were independently extracted by two investigators (NP and EL) and entered into an Excel 2010 (Microsoft Corp., Redmond, WA, USA) spreadsheet. Any discrepancies regarding individual study inclusion, data extraction, and interpretation were resolved by consulting a third investigator (SB). We extracted the following data: first author name, year of publication, number of patients, mean age, and gender of patients. Further we extracted data on the HBV serological status of patients, medical conditions for which patients were treated, the biologic agent used, the presence of other disease modifying antirheumatic drugs (DMARDs), and antiviral prophylaxis. The main outcome used in the meta-analysis was the prevalence of HBV reactivation reported as prevalence proportion. The studies with sample size less than 15 were not included in meta-analysis. Additionally, when available, we collected individual-level data on HBV reactivation, namely, age, gender, condition treated for, anti-TNF agent used, and, if any, antiviral prophylaxis. Based on this data we performed additional meta-analyses according to diseases and biologic agents when possible. Based on the serological status, we stratified results of the meta-analysis according to two main subgroups of patients expected to have different prevalence HBV reactivation: patients presenting with overt chronic HBV infection (being HBsAg positive) and patients presenting with occult HBV infection (being HBsAg negative and anti-HBc positive). Based on information reported in the article full text and tables, it was always possible to obtain the data on the subgroups.

### 2.4. Statistical Analysis

To determine pooled proportions, the variances of the raw proportions (*r*/*n*) were stabilized using a Freeman-Tukey-type arcsine square root transformation [22]: *y* = arcsine[√(*r*/(*n* + 1))] + arcsine[√(*r* + 1)/(*n* + 1)], with a variance of 1/(*n* + 1), where *n* is the denominator for population size. Pooled proportions from all studies were calculated as the back transform of the weighted mean of the transformed proportions, using a random-effects model [23]. We assessed heterogeneity among studies using the Cochran Q test and quantified inconsistencies across studies and their impact on the analysis by using the *I* ^2^ statistic [24, 25]. As we anticipated large heterogeneity considering the very different clinical presentation of patients, we considered statistically significant heterogeneity when *P*_heterogeneity_ < 0.1. The robustness of pooled proportions was explored by conducting sensitivity and subgroup analyses. The meta-analysis was conducted using Stata software (Stata Corp. 2011. Stata Statistical Software: Release 12. College Station, TX: Stata Corp LP).

## 3. Results

**Figure 1:**
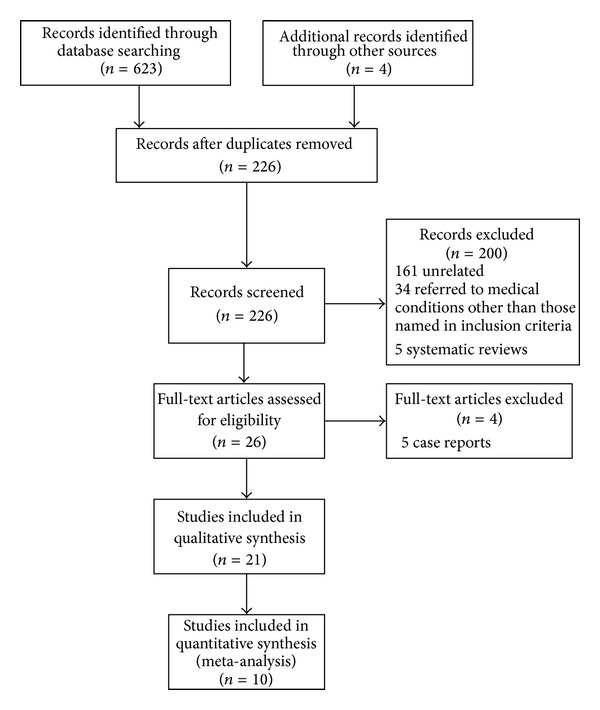
Systematic review and meta-analysis flow chart.

The results of the literature search are reported in a flow chart (Figure 1). After searching 3 databases we identified 632 relevant articles. Additionally, 4 more relevant papers were identified through reference search of relevant systematic reviews. After removing duplicates, 226 remaining abstracts were examined, and further 161 were excluded because of being unrelated to the subject, 34 were excluded because they referred to medical conditions other than those named in inclusion criteria, and 5 were systematic reviews or meta-analyses. Finally when we retrieved full text of the remaining 26 papers, 5 were excluded as they were case reports.

The characteristics of the 21 studies included in the systematic review are reported in Table 1 [11–20, 26–36]. Six studies included patients with overt chronic HBV infection and 9 included patients with occult HBV infection, while 6 studies addressed both groups of patients. Fourteen studies included patients treated with biologic agents because of rheumatoid arthritis (66.7%), 6 (28.6%) patients treated because of ankylosing spondylitis, 5 (23.8%) because of psoriasis, and 4 (19.0%) because of psoriatic arthritis. In 18 (85.7%) studies patients were treated with etanercept, in 16 (76.2%) with infliximab, in 17 (81.0%) with adalimumab, and in 3 (14.3%) with rituximab. In eleven (52.4%) studies antiviral prophylaxis was administered at least to some of patients prior to treatment with biologic agents.

### 3.1. Meta-Analysis

Eleven studies (57.1%) out of 21 were excluded because of the small sample size (<15 subjects) [37], so the analysis was restricted to 10 studies. The result of the meta-analysis is reported in Table 2, with a pooled estimate of HBV reactivation being 4.2% (95% CI: 1.4–8.2%, *I* ^2^: 74.7%), with significant heterogeneity among studies. In the subgroup analyses, the pooled prevalence of HBV reactivation among patients with occult infection was 3.0% (95% CI: 0.6–7.2, *I* ^2^: 77.1%) and the pooled prevalence of HBV reactivation among patients with overt HBV infection was 15.4% (95% CI: 1.2–41.2%, *I* ^2^: 79.9%). In both subgroups, high heterogeneity was present (Table 2).

By restricting the meta-analysis to patients with rheumatoid arthritis the pooled prevalence of HBV reactivation was 3.3% (95% CI: 0.7–7.5%, *I* ^2^: 62.6%), with significant heterogeneity among studies (Table 3). When these results were stratified according to occult or overt infection, results show that the HBV reactivation for patients with occult infection was 2.6% (95% CI: 0.4–6.6%, *I* ^2^: 59.2%), compared with 10.7% (95% CI: 1.4–50.2%, *I* ^2^: 88.8%) among patients with overt chronic infection (Table 3).

We also addressed the prevalence of HBV reactivation in relation to the anti-TNF agent used. The pooled prevalence of HBV reactivation among patients treated with etanercept was 3.9% (95% CI: 1.1–8.4%, *I* ^2^: 51.1%) and 3.0% (95% CI: 0.5–7.6%, *I* ^2^: 49.6%) for those with occult HBV infection (Table 4). Patients treated with adalimumab showed a pooled prevalence of HBV reactivation of 4.6% (95% CI: 0.5–12.5%, *I* ^2^: 28.7%) (Table 5). No case of HBV reactivation was recorded in studies eligible for meta-analysis among 81 patients treated with infliximab (80 occult carriers and 1 overt). Only one patient was treated with rituximab in studies eligible for meta-analysis.

Finally we pooled data on HBV reactivation rate in relation to usage of antiviral prophylaxis. Pooled HBV reactivation rate was 4.0% (95% CI: 1.2–8.3%, *I* ^2^: 75.6%) for patients without any antiviral prophylaxis (Table 6). Analysis on patients submitted to antiviral prophylaxis as well as stratification on occult and overt carriers was not possible because small numbers of cases were included.

## 4. Discussion

Based on meta-analysis we conducted, we report relatively low pooled prevalence of HBV reactivation in patients treated with anti-TNF-*α* agents for rheumatic and dermatological conditions. The pooled reactivation rate for all patients included, as well as for those with RA, was several times higher in chronic overt HBV carriers compared to occult carriers. The pooled reactivations rates for those treated with etanercept and adalimumab were similar and also similar to overall pooled reactivation rate. The pooled reactivation rate for patients with no antiviral prophylaxis did not differ from overall pooled reactivation rate.

Several studies addressed the issue whether anti-TNF-*α* agent induced HBV reactivation, with inconsistent results [10]. We report considerably low overall pooled HBV reactivation rate of 4.2% compared to some authors before, who included both overt and occult patients with reported reactivation rate up to 6.8% [15]. Having this in mind, it could be said that some of previous studies overestimated the risk of HBV reactivation in patients treated for rheumatic and dermatological conditions. This is especially true for the patients with occult infection as Kim et al. [14] and Urata et al. [19] previously reported HBV reactivation rate among occult carriers of 15.6% and 9.6%, respectively, while pooled rate for occult carriers from our analysis is only 3.0%. However, when interpreting these results, substantial heterogeneity among the studies should be taken into account. For example, study by Kim et al. [14] used a 2-fold or greater increase in liver function test as the criteria for reactivation, while most of the others tried to detect HBV DNA. Furthermore regional differences in HBV infection prevalence could also influence the observed reactivation rates because of small samples size. It is possible that presence of certain comorbidities, such as diabetes mellitus or obesity, or some other clinical parameters mediate the risk for HBV reactivation. However no study reported these data so we hope that studies to come will be more informative.

We observed a higher prevalence of HBV reactivation among patients with chronic infection compared to those with the occult one. This is reasonable and expected, as patients positive with anti Hbc antibodies and negative with HBsAg do not necessary harbor undetected intracellular HBV replication. However, the high pooled reactivation rate among overt carriers should be having implication on clinical guidelines, as we consider patients with detectable HBsAg to be eligible for antiviral prophylaxis in order to prevent anti-TNF-*α* induced HBV reactivation. Unfortunately, we were not able to confirm this in our meta-analysis as numbers of patients included were too few to distinguish between the overt carriers subjected and not subjected to antiviral prophylaxis. Instead we were able to calculate only the pooled reactivation rate for all patients nontaking antiviral prophylaxis, irrespective to their HBV infection status. This rate did not differ significantly from the overall pooled one, but the vast majority of cases included were the occult carriers. The future studies including considerable numbers are needed in order to clearly define the criteria for antiviral prophylaxis in patients treated with anti-TNF-*α* agents at risk for HBV reactivation.

We also tried to estimate whether HBV reactivation rate is dependent on anti-TNF-*α* agents used, as well as on underlying condition. However it was possible only to conduct subanalysis on patients treated with etanercept and adalimumab, as on those treated for RA. As observed pooled reactivation rates did not show considerable difference among the subgroups; we did not find any of these to determine patients' susceptibility towards HBV reactivation. Still, if such an association exists it needs to be confirmed in studies to come.

Our meta-analysis has some limitations. We have included only patients treated with anti-TNF-*α* agents for rheumatic and dermatological conditions. This limits external validity of our findings, as anti-TNF-*α* agents are also used for treatment of other autoimmune conditions, such as inflammatory bowel diseases, and are part of chemotherapeutic protocols in treatment of B-cell lymphomas. However, with limiting the analysis on specific subgroup of patients we aimed to decrease heterogeneity and therefore make the results more reliable. Further, we believe that certain comorbidities, such as diabetes and obesity, could mediate a risk for HBV reactivation among patients treated with anti-TNF-*α* agents. Unfortunately none of the studies reported them, so we were not able to include them in the analysis. We were also unable to conduct subanalysis on patients treated with infliximab and rituximab and to distinguish the pooled reactivation rates among overt and occult carriers treated with antiviral prophylaxis. Finally, there is a question of heterogeneity among studies in relation to criteria defining HBV reactivation, which could lead to differences in interpretation of the collected data. Nevertheless, we find our results valuable to the clinicians encountering patients in risk for HBV reactivation being in need for anti-TNF-*α* agents in everyday practice.

In conclusion, although HBV reactivation rate appears to be relatively low in patients treated with anti-TNF-*α* agents for rheumatic and dermatological conditions, the antiviral prophylaxis is recommended in patients with overt chronic HBV infection. More informative studies including large number of cases are needed in order to identify if any patient or treatment related factor mediates the reactivation risk. The individual approach and close monitoring of each patient could be an answer in balancing the need for therapy with hazard associated with HBV reactivation.

## Figures and Tables

**Table 1:** 

Source	Study design	Patients' HBV status	Number of cases^@^	Age, y^#^	Male, %	Followup	Pathological condition	Biologic agent	Antiviral prophylaxis, *n*	Other DMARDs used
Roux et al., 2006 [26]	RS	Overt infection	3	52	100%	nr	RA	ETA, INF, ADA	Lamivudine (3)	MTX (33.3%)

Zingarelli et al., 2008 [27]	PR	Overt infection Occult infection	4	63	25%	19 months^§^	RA	ETA, INF, ADA, RIT	Lamivudine (4)	MTX (25%); COR (25%); HCL (25%); SSZ (25%)

Charpin et al., 2009 [13]	PR	Occult infection	21	58	38%	27 months^#^	RA, AS, PsA	ETA, INF, ADA	No	MTX (47.6%); COR (23.8%)

Chung et al., 2009 [28]	RS	Occult infection	8	37	63%	nr	RA, AS	ETA, INF, ADA	No	MTX (37.5%); SSZ (50%);

Caporali et al., 2010 [11]	PR	Occult infection	67	57	55%	45.5 months^#^	RA, AS, PsA	ETA, INF, ADA	No	MTX (76.1%); COR (64.1%); NSAID (77.6%);

Fotiadou et al., 2011 [29]	RS	Overt infection	7	51	43%	6–24 months	Ps	ETA, INF, ADA	Lamivudine (7)	nr

Kim et al., 2010 [14]	RS	Occult infection	88	51	51%	nr	RA, AS, PsA	ETA, INF, ADA	no	nr

Prignano et al., 2011 [30]	RS	Occult infection	12	62	75%	6 months^#^	Ps	ETA, ADA	no	nr

Cassano et al., 2011 [12]	RS	Occult infection	62	54	68%	nr	Ps	ETA, INF, ADA	no	nr

Kato et al., 2011 [31]	PR	Occult infection	6	62∗	23%∗	8–124 weeks∗	RA	ETA, INF, RIT	no	MTX (2.9%); COR (60%); CYC (42.9%); TAC (17.1%); CYA (2.9%)∗

Lan et al., 2011 [15]	RS	Overt infection Occult infection	88	50.1	13%	nr	RA	ETA, ADA	Lamivudine (10)	MTX (90.9%)

Mori, 2011 [16]	PR	Overt infection Occult infection	32	73^§∗^	37%∗	nr	RA	ETA, INF, ADA	Entecavir (1)	MTX (90%); COR (63.3%); TAC (50%); TOC (8.3%)∗

Tamori et al., 2011 [18]	PR	Overt infection Occult infection	44	59∗	18%∗	24 months	RA	ETA, INF, ADA	Entecavir (2)	MTX (63.3%)∗

Urata et al., 2011 [19]	PR	Occult infection	52	nr	nr	12 months	RA	ETA, INF, ADA, RIT	No	MTX (48.2%); COR (38.5%); SSZ (20.7%); CYC (0.7%); TAC (6.7%); CYA (0.7%); TOC (3.0%); LEF (2.2%); BUC (20%)∗

Cho et al., 2012 [32]	RS	Overt infection	7	43	86%	29 months	Ps	ETA, ADA	Lamivudine (1)	nr

Ryu et al., 2012 [17]	RS	Overt infection	49	43	61%	nr	RA, AS	ETA, INF, ADA	Lamivudine (15) Entecavir (5)	MTX (32.7%); COR (61.2%); HCL (14.3%); LEF (12.2%)

Giardina et al., 2013 [33]	PR	Overt infection Occult infection	11	57^∧^	60%^∧^	24 months^#∧^	RA, AS, PsA	ETA, IFX	Lamivudine (4)	MTX (42.1%)^∧^; COR (59.6%)^∧^

Laurenti et al., 2013 [34]	RS	Overt infection; Occult infection	8	54	50%	nr	PsA	ADA	Lamivudine (1)	nr

Navarro et al., 2013 [35]	RS	Overt infection	4	42	50%	25 months^#∗^	Ps	ETA, INF	Lamivudine (3), Entecavir (1), Adefovir (1)	nr

Nishida et al., 2013 [36]	RS	Overt infection	1	60	100%	47	RA	INF	No	nr

Vassilopoulos et al., 2010 [20]	PR	Overt infection Occult infection	33	52^∧^	38%^∧^	24 months^∧^	RA, AS, PsA	ETA, INF, ADA	Lamivudine (11), Entecavir (3), Tenfovir (1), Telbivudine (1)	nr

nr: not reported. Study design: RS: retrospective cohort; PR: prospective cohort. Pathological condition: RA: rheumatoid arthritis; AS: ankylosing spondylitis; PsA: psoriatic arthritis; Ps: psoriasis. Biologic agent: ETA: etanercept; INF: infliximab; ADA: adalimumab; RIT: rituximab. Other DMARDs: MTX: methotrexate; COR: corticosteroids; HCL: hydroxychloroquine; SSZ: sulfasalazine; NSAID: nonsteroid anti-inflammatory drug; CYC: cyclophosphamide; TAC: tacrolimus; CYA: cyclosporin A; TOC: tocilizumab; LEF: leflunomide; BUC: bucillamine.

^@^Cases included in the analysis.

^#^Expressed as mean.

^§^Expressed as median.

∗Data reported for all patients included in study, not just for those treated with anti-TNF agents.

^∧^Data reported for all patients included in study, not just for those with occult/overt HBV infection.

**Table:** (a)

First author, year	Number of patients	HBV reactivation (*n*)	HBV reactivation (%)	CI 95%	Weight (%)
*All patients *					
Caporali et al., 2010 [11]	67	0	0.0	0.0–5.4	10.9
Cassano et al., 2011 [12]	62	0	0.0	0.0–5.8	10.7
Charpin et al., 2009 [13]	21	0	0.0	0.0–15.5	7.5
Kim et al., 2010 [14]	88	14	15.9	9.7–24.9	11.4
Lan et al., 2011 [15]	88	6	6.8	3.2–14.1	11.4
Mori, 2011 [16]	32	1	3.1	0.6–16.2	8.9
Ryu et al., 2012 [17]	49	3	6.1	2.1–16.5	10.1
Tamori et al., 2011 [18]	44	0	0.0	0.0–7.1	9.8
Urata et al., 2011 [19]	52	5	9.6	4.2–20.6	10.3
Vassilopoulos et al., 2010 [20]	33	1	3.0	0.5–15.3	9.0
Pooled estimate			**4.2**	**1.4**–**8.2**	**100.0**

Heterogeneity chi-squared = 35.55 (d.f. = 9) *P* = 0.000.

*I*-squared (variation in ES attributable to heterogeneity) = 74.7%.

**Table:** (b)

First author, year	Number of patients	HBV reactivation (*n*)	HBV reactivation (%)	CI 95%	Weight (%)
*Patients with overt chronic HBV infection *					
Lan et al., 2011 [15]	18	5	27.8	12.5–50.9	45.5
Ryu et al., 2012 [17]	49	3	6.1	2.1–16.5	54.5
Pooled estimate			**15.4**	**1.2–41.2**	**100.0**

Heterogeneity chi-squared = 4.98 (d.f. = 1) *P* = 0.026.

*I*-squared (variation in ES attributable to heterogeneity) = 79.9%.

**Table:** (c)

First author, year	Number of patients	HBV reactivation (*n*)	HBV reactivation (%)	CI 95%	Weight (%)
*Patients with occult HBV infection *					
Caporali et al., 2010 [11]	67	0	0.0	0.0–5.4	12.3
Cassano et al., 2011 [12]	62	0	0.0	0.0–5.8	12.1
Charpin et al., 2009 [13]	21	0	0.0	0.0–15.5	8.9
Kim et al., 2010 [14]	88	14	15.9	9.7–24.9	12.8
Lan et al., 2011 [15]	70	1	1.4	0.2–7.7	12.4
Mori, 2011 [16]	31	1	3.2	0.6–16.2	10.2
Tamori et al., 2011 [18]	42	0	0.0	0.0–8.4	11.3
Urata et al., 2011 [19]	52	5	9.6	5.4–23.0	11.7
Vassilopoulos et al., 2010 [20]	19	0	0.0	0.0–16.8	8.6
Pooled estimate			**3.0**	**0.6**–**7.2**	**100.0**

Heterogeneity chi-squared = 34.94 (d.f. = 8) *P* = 0.000.

*I*-squared (variation in ES attributable to heterogeneity) = 77.1%.

**Table:** (a)

First author, year	Number of patients	HBV reactivation (*n*)	HBV reactivation (%)	CI 95%	Weight (%)
*All patients *					
Caporali et al., 2010 [11]	59	0	0.0	0.0–6.1	18.4
Lan et al., 2011 [15]	88	6	6.8	3.2–14.1	20.6
Mori, 2011 [16]	32	1	3.1	0.5–15.7	14.5
Ryu et al., 2012 [17]	22	0	0.0	0.0–14.9	12.1
Tamori et al., 2011 [18]	44	0	0.0	0.0–8.0	16.6
Urata et al., 2011 [19]	52	5	9.6	4.2–20.6	17.7
Pooled estimate			**3.3**	**0.7**–**7.5**	**100.0**

Heterogeneity chi-squared = 13.37 (d.f. = 5) *P* = 0.020.

*I*-squared (variation in ES attributable to heterogeneity) = 62.6%.

**Table:** (b)

First author, year	Number of patients	HBV reactivation (*n*)	HBV reactivation (%)	CI 95%	Weight (%)
*Patients with overt chronic HBV infection *					
Lan et al., 2011 [15]	18	5	27.8	12.5–50.9	49.5
Ryu et al., 2012 [17]	22	0	0.0	0.0–14.9	50.5
Pooled estimate			**10.7**	**1.4**–**50.2**	**100.0**

Heterogeneity chi-squared = 8.91 (d.f. = 1) *P* = 0.003.

*I*-squared (variation in ES attributable to heterogeneity) = 88.8%.

**Table:** (c)

First author, year	Number of patients	HBV reactivation (*n*)	HBV reactivation (%)	CI 95%	Weight (%)
*Patients with occult HBV infection *					
Caporali et al., 2010 [11]	59	0	0.0	0.0–6.1	21.5
Lan et al., 2011 [15]	70	1	1.4	0.2–7.7	22.8
Mori, 2011 [16]	31	1	3.2	0.6–16.2	16.3
Tamori et al., 2011 [18]	42	0	0.0	0.0–8.4	18.8
Urata et al., 2011 [19]	52	5	9.6	4.2–20.6	20.5
Pooled estimate			**2.6**	**0.4–6.6**	**100.0**

Heterogeneity chi-squared = 9.80 (d.f. = 4) *P* = 0.044.

*I*-squared (variation in ES attributable to heterogeneity) = 59.2%.

**Table:** (a)

First author, year	Number of patients	HBV reactivation (*n*)	HBV reactivation (%)	CI 95%	Weight (%)
*All patients *					
Caporali et al., 2010 [11]	23	0	0.0	0.0–14.3	12.4
Cassano et al., 2011 [12]	44	0	0.0	0.0–8.0	16.9
Lan et al., 2011 [15]	40	3	7.5	2.6–19.9	16.2
Mori, 2011 [16]	19	0	0.0	0.0–16.8	11.2
Ryu et al., 2012 [17]	38	2	5.3	1.5–17.3	15.9
Tamori et al., 2011 [18]	20	0	0.0	0.0–16.1	11.5
Urata et al., 2011 [19]	38	5	13.2	5.7–27.3	15.9
Pooled estimate			**3.9**	**1.1**–**8.4**	**100.0**

Heterogeneity chi-squared = 12.26 (d.f. = 6) *P* = 0.056.

*I*-squared (variation in ES attributable to heterogeneity) = 51.1%.

**Table:** (b)

First author, year	Number of patients	HBV reactivation (*n*)	HBV reactivation (%)	CI 95%	Weight (%)
*Patients with occult HBV infection *					
Caporali et al., 2010 [11]	23	0	0.0	0.0–14.3	15.3
Cassano et al., 2011 [12]	44	0	0.0	0.0–8.0	20.6
Lan et al., 2011 [15]	31	1	3.2	0.6–16.2	17.7
Mori, 2011 [16]	18	0	0.0	0.0–17.6	13.3
Tamori et al., 2011 [18]	19	0	0.0	0.0–16.8	13.7
Urata et al., 2011 [19]	38	5	13.2	5.7–27.3	19.4
Pooled estimate			**3.0**	**0.5–7.6**	**100.0**

Heterogeneity chi-squared = 9.93 (d.f. = 5) *P* = 0.077.

*I*-squared (variation in ES attributable to heterogeneity) = 49.6%.

**Table 5:** Meta-analysis on HBV reactivation among patients treated with adalimumab.

First author, year	Number of patients	HBV reactivation (*n*)	HBV reactivation (%)	CI 95%	Weight (%)
*All patients *					
Caporali et al., 2010 [11]	19	0	0.0	0.0–16.8	35.0
Cassano et al., 2011 [12]	48	3	0.0	2.2–16.8	65.0
Pooled estimate			**4.6**	**0.5–12.5**	**100.0**

Heterogeneity chi-squared = 1.40 (d.f. = 1) *P* = 0.236.

*I*-squared (variation in ES attributable to heterogeneity) = 28.7%.

**Table 6:** Meta-analysis on HBV reactivation among patients with no antiviral prophylaxis.

First author, year	Number of patients	HBV reactivation (*n*)	HBV reactivation (%)	CI 95%	Weight (%)
*All patients *					
Caporali et al., 2010 [11]	67	0	0.0	0.0–5.4	11.2
Cassano et al., 2011 [12]	62	0	0.0	0.0–5.8	11.0
Charpin et al., 2009 [13]	21	0	0.0	0.0–15.5	8.0
Kim et al., 2010 [14]	88	14	15.9	9.7–24.9	11.7
Lan et al., 2011 [15]	78	6	7.7	3.6–15.8	11.5
Mori, 2011 [16]	32	1	3.1	0.5–15.7	9.3
Ryu et al., 2012 [17]	29	2	6.9	1.9–22.0	9.0
Tamori et al., 2011 [18]	42	0	0.0	0.0–8.4	10.1
Urata et al., 2011 [19]	52	5	9.6	4.2–20.6	7.7
Vassilopoulos et al., 2010 [20]	19	0	0.0	0.0–16.8	10.6
Pooled estimate			**4.0**	**1.2**–**8.3**	**100.0**

Heterogeneity chi-squared = 36.92 (d.f. = 9) *P* = 0.000.

*I*-squared (variation in ES attributable to heterogeneity) = 75.6%.
